# Molecular Analysis of *katG* Encoding Catalase-Peroxidase from Clinical Isolate of Isoniazid-Resistant Mycobacterium tuberculosis

**Published:** 2018

**Authors:** P Purkan, I Ihsanawati, D Natalia, YM Syah, DS Retnoningrum, I Siswanto

**Affiliations:** * Department of Chemistry, Faculty of Sciences and Technology, Airlangga University; Surabaya, Indonesia; ** Biochemistry Research Division, Faculty of Mathematics and Natural Sciences, Bandung Institute of Technology, Bandung, Indonesia; *** School of Pharmacy, Bandung Institute of Technology, Bandung, Indonesia

**Keywords:** isoniazid, Mycobacterium tuberculosis, katG, catalase-peroxidase

## Abstract

Isoniazid (INH) is a drug for the treatment of tuberculosis in patients infected with Mycobacterium tuberculosis. The katG enzyme, or catalase-peroxidase, activates the pro-drug INH that is coded by the katG gene in M. tuberculosis. Mutations of the katG gene in M. tuberculosis are a major INH resistance mechanism. The M. tuberculosis clinical isolate R2 showed INH resistance at a high level of 10 µg/mL. However, the molecular basis for the resistance is unclear. The identification of a mutation in the katG gene of the clinical isolate R2 showed four mutations, i.e., C1061T, G1261 A, G1388T, G2161A, which correspond to the amino acid substitutions T354I, G421S, R463L, and V721M, respectively. The mutant katG gene, along with the wild-type were cloned, expressed and purified. The mutant enzyme showed 86.5% of catalase and 45% of peroxidase activities in comparison to the wild type. The substitutions of T_354_I and G_421_S in mutant katG R2 created significant instability in the adduct triad complex (Trp107-Tyr229-Met255), a part of the active site of the catalase-peroxidase enzyme in the model structure analysis. The events could be based on the high resistance of the clinical isolate R2 toward INH as the molecular basis.

## Introduction

Isoniazid (isonicotinic acid hydrazide, INH) is a prodrug which forms a key part of the frontline chemotherapy used to treat tuberculosis (TB) in many countries. INH has been widely used to treat the TB disease caused by *Mycobacterium tuberculosis* since it is cost-effective and exhibits a high bactericidal effect [**1**][**2**]. INH has a minimal inhibitory concentration (MIC) to kill *M. tuberculosis* at a dosage between 0.02 – 0.2 μg/mL [**3**][**4**]. Apart from multidrug-resistant TB (MDR-TB), about 9.5% (8.1% in new and 14.0% in previously treated) of TB cases worldwide in 2017 were estimated to have isoniazid-resistant TB without MDR-TB. This is associated with an increased risk of treatment failure in patients who receive first-line regimens [**1**].

To function as an antitubercular agent, INH requires activation of the catalase-peroxidase enzyme encoded by the *M. tuberculosis* katG gene [**4**]. The INH is bound by catalase-peroxidase in its active site, then converted to an isonicotinoyl acyl radical through the use of a diazene intermediate [**4**]. The isonicotinoyl acyl radical interacts with the NADH electron donor in the active site of the enoyl ACP reductase (InhA) enzyme [**5**]. The NAD-INH complex is known as a potent inhibitor of InhA, the enzyme that has an important role in the biosynthesis of mycolic acid, the cell wall component in mycobacteria [**5**].

The catalase-peroxidase from *M. tuberculosis* (katG) is a homodimer protein with two subunits of 80 kDa. Each subunit has two dominant α-helix domains, which means that the domains originated from gene duplication. The N domain has a heme, an active site and a substrate binding site. While the C domain does not have those, its presence is needed to support the overall enzyme activity [**6**] [**7**] [**8**]. The catalytic activity of katG is mediated by some residues in the active site that resided around the heme group. The heme is surrounded by six residues which are Arg-104, Trp-107 and His-108 in the distal pocket, and His270, Trp321 and Asp381 in the proximal pocket. In the heme, the Trp107 residue is connected to Tyr229 and Met255 residues to form an adduct triad complex. The adduct triad is likely conserved in many catalase-peroxidase structures and it is involved in the catalase activity [**9**]. The binding of INH to katG takes place at the edges of the δ-meso heme. In the region, the residues of the distal pocket, i.e., Arg104, Trp107 and His108, are involved in the interactions with INH [**9**].

Mutations in katG that change catalase-peroxidase activities are generally associated with INH resistance in *M. tuberculosis*. The strain of *M. tuberculosis* which has a genetic deletion of katG or mutation acquires resistance to INH [**6**][**10**]. Around 60-70% of INH-resistant *M. tuberculosis* has mutations in katG and the remainder has mutations in *inhA, ahpC* and *kasA* genes [**6**] [**11**]. A structure-activity study showed that the resistant mutant katG (S315T) still has 50% of catalase-peroxidase activities [**12**][**13**]. Even though katG (S315T) has catalase and peroxidase activities, it is less efficient than KatGWT in the isoniazid metabolism [**14**]. Modification of the INH binding site due to the S315T mutation is a significant factor in the decline of the mutant activity to activate isoniazid [**15**] [**16**].

The continued rise in drug-resistant and multidrug-resistant strains of TB and the scale of the TB epidemic have stimulated fundamental research to elucidate the molecular mechanisms of anti-TB drugs, including INH, hoping that this information can initiate the discovery of new antimicrobial targets and alternative treatment regimes. A clinical isolate R2 of *M. tuberculosis* showed a high INH resistance at 10 µg/mL; however, the molecular basis for the resistance is unclear. The paper reported the molecular analysis of katG mutation in the R2 isolate, and activity and structure analysis of its protein to solve the INH resistance in the isolate.

## Material and Methods

**Plasmids and bacterial strains**

The pT7Blue and pCold II DNA plasmids were used as cloning and expression vectors, respectively. The INH sensitive *M. tuberculosis* (H37RV) was obtained as an ATCC strain, while the INH-resistant *M. tuberculosis* (R2) was obtained from the Pulmonary Hospital in Bandung - Indonesia.

**The bacterial culturing**

The *M. tuberculosis* was grown in Löwenstein-Jesen (LJ) medium [**10**] containing 60% (w/v) egg suspension, 1% (w/v) malachite green, 0.8% (v/v) glycerol, 0.2% (w/v) KH_2_PO_4_, 0.02% (w/v) MgSO_4_.7H_2_O, and 0.04% (w/v) C_6_H_6_MgO_2_, then incubated at 37°C for a week, whereas the *E. coli* was grown in Luria-Bertani (LB) medium at 37°C for 16 hours. The LB is composed of 0,5% (w/v) yeast, 1% (w/v) NaCl, and 1% (w/v) tryptone. For a solid medium, 2% (w/v) bacto agar was added. The recombinant *E. coli* was grown in LB medium containing 100 µg/mL ampicillin.

**Amplification of katG**

The DNA template for PCR was prepared by treating the mycobacterium cells in lysis buffer (5 mM TrisCl, pH 8.5; 0.1 mM EDTA pH 8.5; and 0,5% (w/v) tween-20) containing 0.2 mg/mL proteinase K, and incubating at 50°C for 1 hour, then at 95°C for 3 minutes. The supernatant which contains DNA was separated from the debris cells by centrifugation at 11.000g for 3 minutes.

The total volume for PCR was 25 µL and it was composed of 5 µL DNA template (25 ng DNA); 20 pmol of each primer; 1.25 units of *Taq DNA polymerase* (Roche); buffer 1x (10 mM Tris HCl pH 9; 1.5 mM MgCl_2_; 50 mM KCl) and 100 µM of dNTP mix (Roche). The FG primer is composed of nucleotides 5’- CATATGAAATACCCCGTCGAGGGCG-3’ containing *Nde*I restriction site in 5’ terminal, whereas the RG primer has a nucleotide sequence of 5’- TCTAGATCAGCGCACGTCGAACCTGTC-3’ containing *Xba*I restriction site in 5’ terminal. The process was performed by a gene cycler machine (Bio-Rad, Germany) in 25 cycles, where each cycle was set at 94°C (1 minute) for denaturation, 54°C (1 minute) for annealing and 72°C (3 minutes) for polymerization. The PCR process was initiated by a pre-denaturation step at 94°C for 5 minutes, and finished by a post extension step at of 72°C for 7 minutes.

**The cloning of katG**

After being purified with a GFX purification kit (Amersham), The PCR product corresponding to katG was inserted into the pT7Blue vector with T4 DNA ligase (Roche). The product was then used to transform *E. coli* DH5α which had previously been treated with 100 mM CaCl_2_ at low temperature [**17**]. The transforming cells were screened in solid LB containing 100 µg/µL ampicillin, 0.1µM isopropyl β-D-1-thiogalactopyranoside (IPTG) and 0.1 µM 5-bromo-4-4-chloro-3-indolyl-β-D-galactopyranoside (X-Gal) [**17**]. The white colonies were screened to get a positive clone carrying recombinant plasmid pT7-katG including a sequencing of katG using the primers listed in **Table 1**. The katG gene was then sub-cloned in pCold II-DNA and expressed in *E. coli* BL21 (*DE3*).

**Table 1:** The nucleotide of primers

No	Primer	Number of nucleotides	Nucleotides of primers (5’→3’)
1	SP6 promoter	24	catacgatttaggtgacactatag
2	T7 promoter	20	taatacgactcactataggg
3	FG (*Nde*I)	32	catatgaaataccccgtcgagggcg-
4	RG (*Xba*I)	32	tctagatcagcgcacgtcgaacctgtc
5	KF	20	gcagatggggctgatctacg
6	FDPRK	18	cgacgagttcgccaaggc
7	*katG*F	28	ggtcatatgaaataccccgtcgagggcg
8	*katG*R	30	cgtctagactcagcgcacgtcgaacctgtc

**Expression of katG protein**

A recombinant of *E. coli* BL21(*DE3*) carrying pCold II-katG was cultured in 10mL LB medium containing 100 μg/mL ampicillin, then incubated at 150 rpm and 37°C for 5-6 hours to obtain an optical density of 0.4-0.5 at λ 600 nm. The culture was then immediately cold shocked at 15°C for 30 minutes without shaking, followed by the addition of 0.1mM isopropyl β-D-1-thiogalactopyranoside (IPTG), and incubating at 150 rpm, 15°C for 24 hours. The cells were harvested by centrifugation at 5.000g at 4°C for 10 minutes. The cells pellet was washed with a lysis buffer (50 mM Tris-Cl, pH 7.4; 200 mM NaCl), then re-centrifugated at 5.000g at 4°C for 10 minutes. The cells pellet was suspended in 10 mL of 0.02 M phosphate buffer at pH 7 and then lysed using pulse sonication for 30 seconds per minute for 10 minutes with a power setting of 4. The supernatant was separated from debris by centrifugation at 10.000g at 4°C for 20 minutes. The katG protein in the supernatant was detected by SDS PAGE [**2**][**21**].

**Purification of katG**

The katG protein was purified with the affinity chromatography technique by using a HisTrap HP column containing a Ni Sepahrose matrix. The protein sample adjusted to pH 7.4 with 0.02 M phosphate buffer contained 25-50 mM NaCl and 10 mM imidazole. Firstly, the column matrix was equilibrated with binding buffer (50 mM NaH_2_PO_4_, pH 7.4, 25 mM NaCl, 10 mM imidazole), followed by pouring the protein sample into the column. The protein was eluted by gradient elution buffer (50 mM NaH_2_PO_4_, pH 7.4, 25-50 mM NaCl) containing 50-200 mM imidazole. Each fraction was collected at 1 mL, then all the fractions were analyzed by SDS PAGE [**2**].

**SDS-PAGE**

The expressed and purified protein was analyzed by SDS PAGE using 12% (w/v) and 4% (w/v) acrylamide on separating and stacking* g*el, respectively. The separation process was run at 120 mV for 1.5 hours [**17**].

**Protein content and Heme assay**

The protein content of the crude lysate and eluate during the purification process were estimated using the Bradford Protein Assay kit with bovine serum albumin (BSA) as a standard. The blue color formation was based on a reaction between the proteins and Coomassie brilliant blue G250 solution that was recorded spectrophotometrically at 595 nm [**18**]. The optical purity of heme-containing protein of katG was determined based on the Unnisa method, by recording the absorbance of the final eluate at 408 nm for the heme protein (KatG) and 280 nm for the total protein [**19**].

**Catalase-peroxidase activities assay**

The catalase activity was assayed based on the Patti and Bonet-Maury method [**20**]. The 12.5 mM H_2_O_2_ substrate reacted with the katG protein in a 10 mM K-phosphate buffer, pH 7.0 with a total volume of 1 ml for 10 min. The mixture was then added 2.5 ml of titanium reagent to stop the enzymatic reaction, then the formed yellow color was observed at λ 410 nm [**20**]. One unit activity of catalase was defined as the amount of enzyme decomposing 1 mmol of H_2_O_2_ per min.

The assay of the peroxidase activity was performed through the reaction of 100 µM O-dianisidine in a 50mM potassium phthalate buffer (pH 4.5) containing 25 mM tert-butyl hydroperoxide (t-BHP) with 12.5mM H_2_O_2_ and katG protein for 10 minutes [**14**]. The product of o-dianisidine quinonediimine from the reaction was detected by spectrophotometry at λ 460 nm (ε_460_ = 11.3mM^-1^ cm^-1^). One unit of peroxidise activity was defined as the amount of enzyme needed to form 1 µmol of product per minute at 30°C.

**Structural modeling, docking and molecular dynamics simulation**

The structure model of mutant katG R2 was constructed with the help of the SWISS-MODEL server, using the known crystal structure of a wild-type katG (PDB code 1SJ2) as a template. The root mean square deviation of the model was compared to the 1SJ2 structure using a superposition server (SuperPose version 10) [**2**] [**21**], then visualized with a PyMOL 1.3 server. A docking simulation was performed by Autodock4 [**22**] using INH as substrate and both ligand and receptor were docked in a rigid state. This docking aimed to find the initial coordinate for the molecular dynamics (MD) simulation. MD simulations were performed with the Amber 16 simulation package program [**23**]. It ran on a PC with the following specification: Intel Core i3 processor, 6 GB RAM on a Linux Ubuntu 16.04.3 operation system. In order to accelerate the simulation process, simulations were run on CUDA [**24**] powered GPU Nvidia BTX 1080Ti 11 GB. Binding energy as a measure of the affinity of ligand to katG structure was calculated using MMGBSA.py methods [**25**].

## Results and Discussion

**The *katG* gene of INH resistant *M. tuberculosis* R2**

In the present study, the selected clinical isolate R2 showed four mutations which were identified using sequence analysis of the katG gene. In order to explore the rationale behind INH resistance in the clinical isolate R2, the mutant katG gene was cloned, expressed and purified, followed by a structural analysis of the katG protein in comparison to the wild-type.

**Fig. 1:**
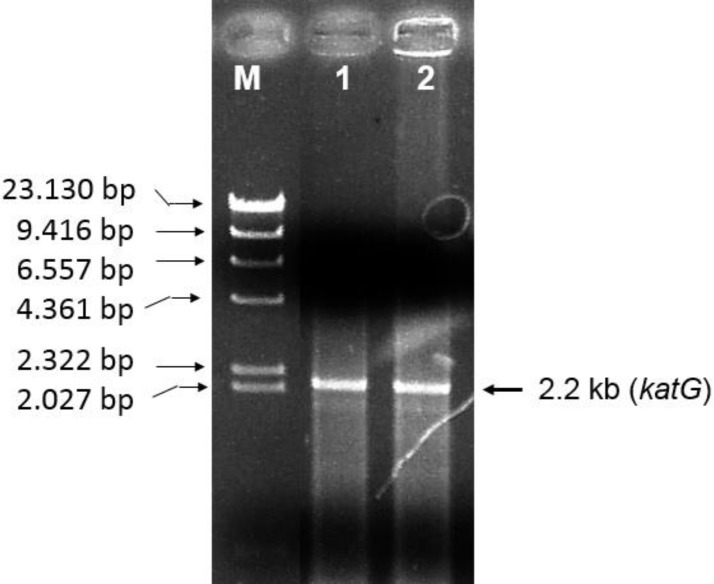
PCR product in agarose gel electrophoresis. M, Marker DNA λ/*Hind*III (M); lane 1 and 2, represented the DNA fragment (2,2 kb) that resulted by PCR using the genome of *M. tuberculosis* H37RV and INH-resistant *M. tuberculosis* R2 as templates respectively. The DNA fragment of 2.2 kb corresponds to the *katG* gene.

**Fig. 2:**
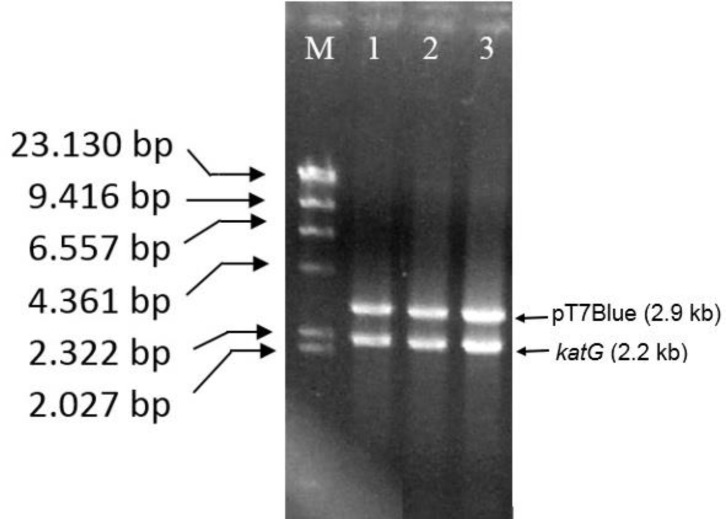
**The pT7-Blue-*katG* recombinant in agarose gel electrophoresis**. M, marker DNA λ/*Hind*III; lane 1, pT7Blue-*katG M. tuberculosis* H37RV/ *Nde*I+*Xba*I; lane 2-3, pT7Blue-*katG* R2/ *Nde*I+*Xba*I. The pT7Blue had a size of 2.9 kb, then *katG* had a size of 2,2 kb.

**Fig. 3:**
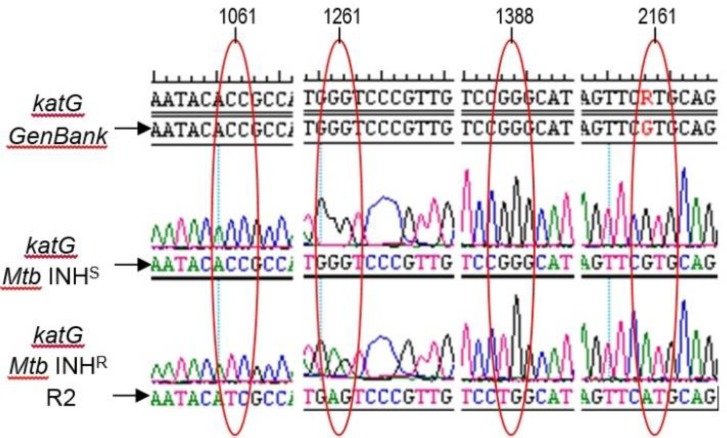
**Electrophoretogram of sequence *katG* R2 toward *katG* H37RV and Genbank.** KatG R2 had four mutations, C_1061_T, G_1261_ A, G_1388_T, G_2161_A compared to katG H37RV.

An amplification of katG from INH-sensitive *M. tuberculosis* (H37RV) and INH-resistant strains (R2) with PCR generated a DNA fragment of 2.2 kb (**Fig 1**),which is attached to the catG in *M. tuberculosis* H37RV in Genbank (ID: 885638). Cloning the DNA fragments in pT7Blue vector could result in the recombinant DNA of 4.1 kb. Digestion of recombinant pT7Blue-*katG* with *Nde*I and *Xba*I enzymes produced two fragments of 2.2 kb and 2. 9 kb (**Fig 2**), corresponding to the katG fragment and pT7Blue vector (2.9 kb), respectively. The katG R2 had 4 mutations compared with the katG wild-type (H37RV), namely C_1061_T, G_1261_ A, G_1388_T, G_2161_A (**Fig 3**), then changed the amino acids of T_354_I, G_421_S, R_463_L, and V_721_M in its protein (**Table 2**). The three mutations of T_354_I, G_421_S, and V_721_M classified as a new type of mutation, which had not been previously found. Mutations of katG in clinical isolates are unique in each geographical area where clinical isolates are found [**3**] [**26**] [**27**]. Profiling the mutations is required to construct a genetic marker in the INH-resistant clinical isolates.

**Table 2:** The profile of katG of INH resistant M. tuberculosis clinical isolate (R2)

Gene	Clinical isolate of *Mtb* INH^R^	Mutation				The level of INH resistance [µg/mL]
		Σ	Nucletide	Σ	Amino acid	
*katG*	R2	4	C _1061_ T G _1261_ A G _1388_ T G _2161_ A	4	T_354_I^a^ G_421_S^a^ R_463_L^b^ V_721_M^a^	10

^*a*^ *new mutations*

^*b*^ *this mutation was also reported by Brossier et al, 2006 (G1388T & G1481A [**27**].*

**The catalase-peroxidase activities and structure of katG R2**

**Fig. 4:**
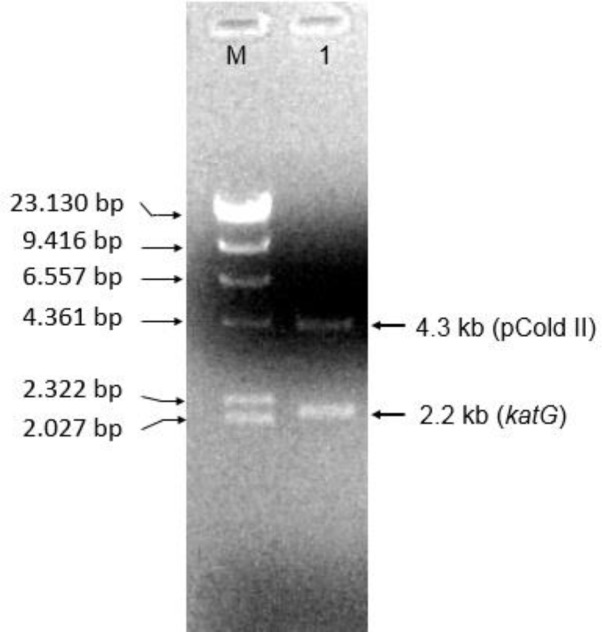
**The pCold II-*katG* recombinant in agarose gel electrophoresis.** M, marker DNA λ/Hind III; 1, restriction of pcold II-*katG* recombinant with *Nde* I and *Xba*I resulted in fragments of 4.2 kb and 2.2 kb belonging to pCold II-DNA and katG respectively.

**Fig. 5:**
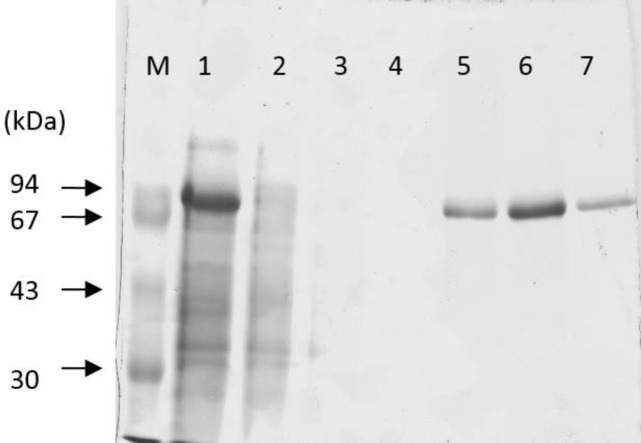
**KatG protein in SDS-PAGE.** M (marker), 1, crude protein of katG; 2, flow throw; 3-4 washing; 5, protein katG WT; 6-7, protein katG R2 after eluted with 150 mM imidazole

An expression of katG was performed in *E.coli* BL21 (*DE3*) using a pCold II-DNA vector. A subclone of katG in pCold II-DNA produced the 6.5 kb DNA fragment which represented a combination of 4.3 kb from pCold II-DNA and 2.2 kb from the katG fragment (**Fig 4**). The expression of the katG gene could produce a protein of 80 kDa based on sodium dodecyl sulphate-polyacrylamide gel electrophoresis analysis (**Fig 5**, lane 1).

**Fig. 6:**
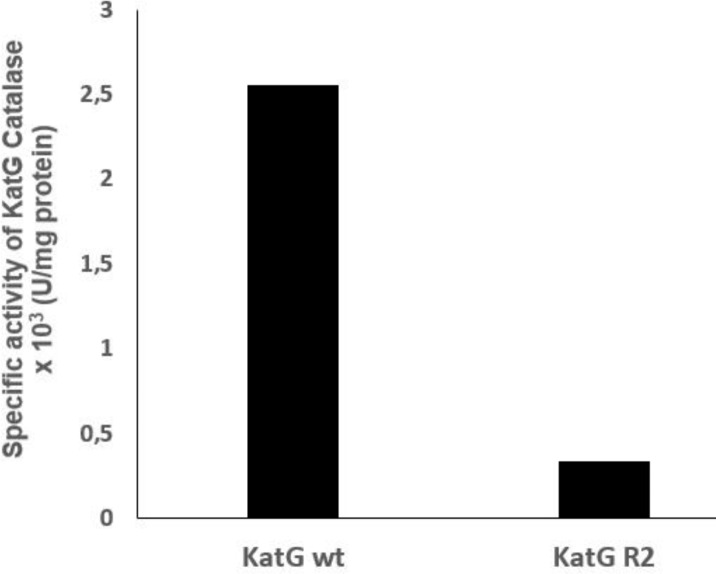
**The catalase activity of katG.** The mutant katG R2 had a catalase activity of 86.5% of the wild-type activity.

**Fig. 7:**
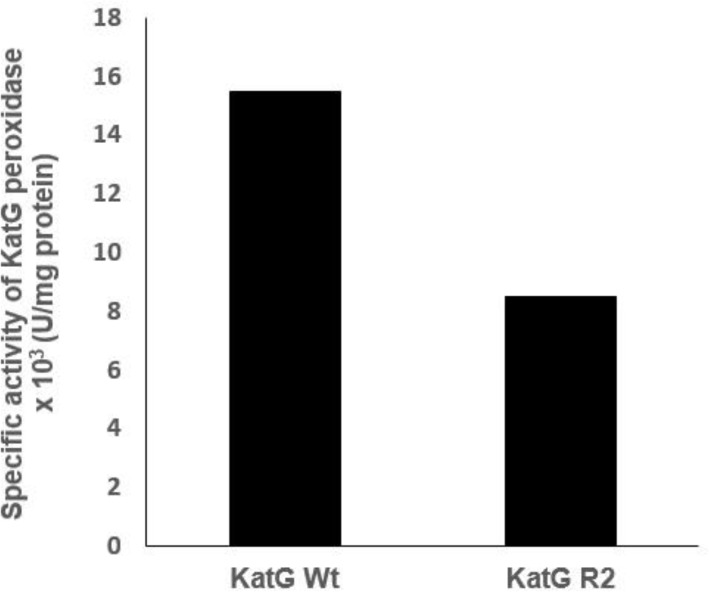
**The peroxidase activity of katG.** The mutant katG R2 had a peroxidase activity of 45% of its wild-type activity.

The purification of the katG protein in a HisTrap HP column with a Ni Sepharose matrix developed a pure protein of 80 kDa in SDS PAGE after it was eluted with 150 mM imidazole (**Fig 5**, lane 5-7). The optical purity ratio of the heme protein of katG in the final eluant which is defined as a ratio of A408/A280 showed 0.71 for the WT and 0.79 for the mutant. The mutant katG R2 exhibited a lower catalase and peroxidase activity than the wild-type katG. The catalase and peroxidase activity of the mutant katG R2 showed 86.5% and 45% respectively from its wild-type activity (**Fig 6** and **7**). The substitution of 4 amino acids in the katG R2 might trigger the decrease of catalase-peroxidase activities in the mutant.

Many mutations in the katG gene have been identified [**23**][**34**], but only a handful are located within the active site of the protein. The remainder appears to be located either on the surface of the protein, where they may play a role in governing katG dimerization (katG is a functional homodimer), or in protein stability [**35**][**36**]. Disruption of hydrogen-bonding networks or electron-transfer pathways may also occur as a result of these mutations.

The structure of mutant katG R2 had been changed with RSMD 0,3 Å toward its wild-type structure. For further analysis, INH-KatG interaction was simulated by docking and molecular dynamic simulation. The wild-type KatG–INH complex and mutant KatG R2–INH complex binding energies were found to be -18.4610 ± 1.5622 and -14.8351 ± 1.4941, respectively (**Table 3**). The data indicate that the INH would be functionally active with the wild-type katG compared to the mutant katG R2.

**Table 3:** Binding energy between INH and katG both WT and R2

Energy Component	Energy (kcal/mole)	
	KatG WT	KatG R2
VDWAALS	-15.8981 ± 1.6596	-18.7344 ± 1.6055
EEL	-33.0993 ± 3.1106	-13.3143 ± 2.8692
EGB	33.3649 ± 1.7621	20.2007 ± 2.2141
ESURF	-2.8284 ± 0.0676	-2.9871 ± 0.0516
DELTA G gas	-48.9975 ± 2.8595	-32.0487 ± 2.7244
DELTA G solv	30.5365 ± 1.7874	17.2136 ± 2.2118
Total binding free energy	**-18.4610 ± 1.5622**	**-14.8351 ± 1.4941**

***Note:*** *VDWAALS = van der Waals force; EEL = electrostatic energy; EGB = electrostatic contribution to the solvation free energy; ESURF = nonpolar contribution to the solvation free energy*

**Fig. 8:**
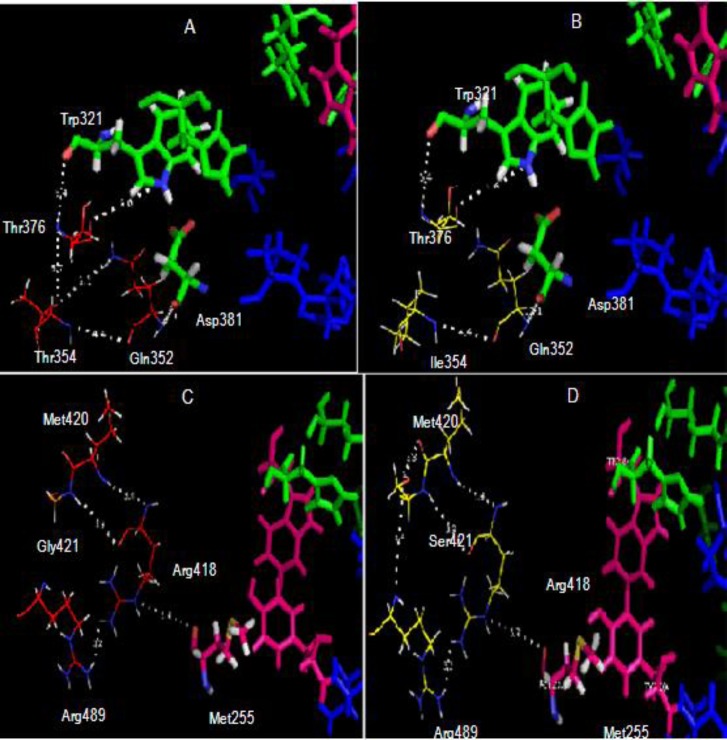
**Illustration of effect Gly421Ser, Arg463Leu substitution in katG R2 structure model.** (A) The wild-type katG structure with Thr354 polar residue in the catalytic site region (green represents amino acid residues). (B) The mutant katG structure L10 that carried Ile354 residue. The Thr354Ile substitution eliminated the interaction between Thr354 and Thr376. (C) The wild-type katG structure with a stable interaction in the adduct triad complex (a magnetized rod-shaped residue of Ser421 residue causing a new interaction linking the Ser421 residue to the Arg489 residue). This new interaction is not found in the katG WT structure.

The mutant katG R2 had several intramolecular interaction changes in its active site region, such as a disruption of Van der Waals interaction between Thr354 residue with Thr 376; one of hydrogen bonds between Thr354 with Gln352 (**Fig 8**, A and B), and the appearance of a new interaction between Ser421 with Arg489 and a hydrogen bond between Ser421 with Met420 (**Fig. 8**.C and D). The mutant also lost Van der Waals interaction between Met255 and Arg418 which laid in the adduct triad region (Trp107-Tyr229-Met255), and the interaction is needed to stabilize the active site of catalase-peroxidase. All interactions change the mutant katG R2 structure which, induced by Thr354Ile and Gly421Ser substitution, might induce the decrease of catalase-peroxidase activities. The R463L alteration is well known as a polymorphism in katG variants including for the katG R2 [**6**] [**11**]. The effect of the alteration on the activity of catalase-peroxidase is negligible, as well as the V721M alteration which is located on the outer surface of structural katG R2. Although the catalase-peroxidase activities and the presence/absence of the Met-Tyr-Trp cross-link declared by Cade and Ghiladi [**37**][**38**] are not associated with the level of INH-resistance phenotype in the katG mutations, the INH-NADH adduct formation as catalyzed by the compound intermediates of katG correlates with isoniazid susceptibility/resistance pathways in TB [**38**]. Alteration of katG function to perfectly form the INH-NADH adduct might facilitate the emergence of high resistance in the clinical isolate R2 to INH.

The correlation of catalase and peroxidase activity among katG variants with the INH resistance level has previously been reported. The mutant katG has five amino acid replacements in the C terminal domain, i.e., L437P, R463L, G494D, I518T, and K554E, and exhibits very low catalase and peroxidase activity linked to the high resistance toward INH [**21**] [**27**] [**30**]. However, the mutant katG (S140N, A350T, R463L, R463G and L587M) which has catalase-peroxidase activities higher than wildtype katG, exhibited INH sensitivity. The mutant katG (S315T) that retained peroxidase and catalase activity as 60% and 40% respectively from its wild-type activity developed INH inhibitory levels to the transformant BCG corresponding to the decline of its protein activity [**13**] [**31**] [**32**].

The interaction model for simulation provides a useful structural framework for designing new antitubercular agents that can circumvent INH resistance [**29**]. Ramasubban et al. studied the MD simulation of mutant katG (His276Met, Gln295His and Ser315His). The mutant had a decreased flexibility at active site residues and unstable backbone conformation compared with WT, which in turn resulted in an impairment of enzyme function to bind INH [**28**]. The mutant katG R2 lost 86.5% of catalase and 45% of peroxidase activities from its wild-type. Of amino acids alteration in the mutant, substitution of T_354_I and G_421_S created significant instability in the *adduct triad* complex (Trp107-Tyr229-Met255), a part of the active site of the catalase-peroxidase enzyme in the model structure analysis. In a dynamic simulation, it was shown that the mutant bound more difficult INH compared to the wild-type katG. Site-directed mutagenesis will be performed in the future to determine the critical residue involved in the decrease of catalase and peroxidase activity of mutant katG R2.

## Conclusion

The molecular basis of INH resistance in a clinical isolate of *M. tuberculosis* R2 showed the katG gene of the isolate had four mutations corresponding to amino acid replacements T354I, G421S, R463L, and V721M on its protein. The mutations were accompanied by a decrease in the catalase-peroxidase activities of katG R2. Of amino acid alterations, substitution of T_354_I and G_421_S created significant instability in the *adduct triad* complex (Trp107-Tyr229-Met255), a part of the active site of the catalase-peroxidase enzyme in the model structure analysis. The events might lead to INH resistance in the clinical isolate R2.

**Acknowledgments**

We gratefully thank the Directorate General of Higher Education, Republic of Indonesia, for funding the research at the Department of Advanced Bioscience, Kinki University, Japan. We would also like to thank Prof. Hunsa Punnapayak, Chulalongkorn University for critically reading the manuscript.
